# A modest start, but a steady rise in research use: a longitudinal study of nurses during the first five years in professional life

**DOI:** 10.1186/1748-5908-7-19

**Published:** 2012-03-19

**Authors:** Lars Wallin, Petter Gustavsson, Anna Ehrenberg, Ann Rudman

**Affiliations:** 1 Department of Neurobiology, Care Sciences and Society, Division of Nursing, Karolinska Institutet, Stockholm, Sweden; 2 Clinical Research Utilization, Karolinska University Hospital, Stockholm, Sweden; 3 Department of Clinical Neuroscience, Karolinska Institutet, Stockholm, Sweden; 4 School of Health and Social Studies, Dalarna University, Falun, Sweden

## Abstract

**Background:**

Newly graduated nurses are faced with a challenging work environment that may impede their ability to provide evidence-based practice. However, little is known about the trajectory of registered nurses' use of research during the first years of professional life. Thus, the aim of the current study was to prospectively examine the extent of nurses' use of research during the first five years after undergraduate education and specifically assess changes over time.

**Method:**

Survey data from a prospective cohort of 1,501 Swedish newly graduated nurses within the national LANE study (Longitudinal Analyses of Nursing Education and Entry in Worklife) were used to investigate perceived use of research over the first five years as a nurse. The dependent variables consisted of three single items assessing instrumental, conceptual, and persuasive research use, where the nurses rated their use on a five-point scale, from 'never' (1) to 'on almost every shift' (5). These data were collected annually and analyzed both descriptively and by longitudinal growth curve analysis.

**Results:**

Instrumental use of research was most frequently reported, closely followed by conceptual use, with persuasive use occurring to a considerably lower extent. The development over time showed a substantial general upward trend, which was most apparent for conceptual use, increasing from a mean of 2.6 at year one to 3.6 at year five (unstandardized slope +0.25). However, the descriptive findings indicated that the increase started only after the second year. Instrumental use had a year one mean of 2.8 and a year five mean of 3.5 (unstandardized slope +0.19), and persuasive use showed a year one mean of 1.7 and a year five mean of 2.0 (unstandardized slope +0.09).

**Conclusion:**

There was a clear trend of increasing research use by nurses during their first five years of practice. The level of the initial ratings also indicated the level of research use in subsequent years. However, it took more than two years of professional development before this increase 'kicked in.' These findings support previous research claiming that newly graduated nurses go through a 'transition shock,' reducing their ability to use research findings in clinical work.

## Background

Healthcare in many countries is facing growing demands from an ageing population, in parallel with decreasing resources. In order to optimize healthcare effectiveness, evidence-based practice has been proposed as a strategy for incorporating new and adequate knowledge into practice [1-3]. The application of the best available evidence in the care of individual patients is in fact crucial for all healthcare organizations to deliver quality care. Evidence-based practice includes consciously applying the best available evidence based on research findings, clinical experience, and patient preferences [1]. Because nurses are the largest group of healthcare practitioners, their contribution to evidence-based practice is pivotal [4]. It is, therefore, disquieting that studies indicate that nurses' use of research in their clinical practice varies considerably [5-7]. Additionally, little is known about the transition from education to practice and how nurses' capacity for making use of research findings in clinical practice develops over time. Thus, this study investigated the extent of Swedish nurses' perceived use of research over the first five years of their professional career.

In order to enhance the readiness for a changing healthcare system, Swedish nursing education has shifted from vocational training to a three-year bachelor's degree program, in parallel with developments in many other countries. This shift involves an academic perspective in both content and educational methods, including courses in research methods, a bachelor's thesis, and a move towards more self-directed learning. Newly graduated nurses are thereby expected to posses the skills underpinning evidence-based practice, namely questioning prevailing practices, searching, critically appraising, and using scientific knowledge in clinical practice [8]. However, research studies and a national audit of higher education show that the academic perspective in education is still deficient and nursing students perceive a gap between the academic and clinical elements of their education [9,10]. These flaws in undergraduate education may impact newly graduated nurses' capacity to base their practice on research findings.

Research utilization is a prominent facet of the concept of evidence-based practice. A definition was proposed by Estabrooks and colleagues: 'that process by which specific research-based knowledge (science) is implemented in practice' [11]. Research utilization has been conceptualized to comprise instrumental, conceptual, and persuasive use of research. The concepts were developed in social science, *e.g*., by Larsen, who proposed that knowledge utilization could be classified as instrumental and conceptual [12]. Beyer and Trice added symbolic (persuasive) utilization [13]. These three research utilization concepts were presented to nursing researchers through the work of Stetler [14,15]. Estabrooks continued this work by developing definitions and measures and assessing the constructs in nursing [16]. Instrumental utilization refers to the concrete application of research to practice in making specific decisions/interventions; conceptual utilization refers to a change in thinking in response to research, but not necessarily in behaviour (an informing and enlightening use); and persuasive utilization refers to the use of specific research findings to convince others.

In a recent systematic review of instruments for measuring research use, Estabrooks' operationalization of instrumental, conceptual, and persuasive use is called 'kinds of research use' [17]. In another systematic review on extent of nurses' use of research, Squires *et al*. report five surveys using 'kinds of research use' as a measure [7]. Research use ranged from moderate-low to high depending on the kind of research use, on average these results represented 5 (using research on half of the shifts) on a 7-point frequency response scale. In Estabrooks' index study, a survey including Canadian staff nurses, scores were highest for conceptual use, followed by instrumental and persuasive use [16]. Profetto-McGrath *et al*. found the same distribution of results among Canadian nurses in adult surgical and paediatric care [18]. Kenny, who investigated nurses in US Army hospitals, also reported a comparable distribution [19]. Milner and co-workers studied research use among Canadian staff nurses, educators and managers, and reported-in line with the other studies-that conceptual use of research was most frequent, with persuasive being least frequent [20]. In contrast, in two recent Swedish studies, including two national samples of newly graduated nurses (one to three years after graduation), instrumental research use was most common, followed by conceptual and persuasive use [6,21]. Aside from one of the Swedish studies [6] that had a one-year longitudinal approach (two measurement waves), we have not been able to identify any study using the 'kinds of research use' measures in a longitudinal study. Looking at studies using other measurement tools for research use, there appear to be a few intervention studies using pre- and post-measurement designs, thus not presenting true longitudinal data having more than two measurement occasions [7].

As in many other developed countries, Swedish healthcare is challenged by increasing demands on the healthcare system and simultaneously being allocated fewer resources. The number of hospital beds has decreased by 21% during the period from 1999 to 2008. Today, Swedish healthcare has the fewest hospital beds per inhabitant compared to other countries within the OECD (Organization for Economic Co-operation and Development). This has resulted in an estimated number of occupied beds in medical wards of 100% to 105% [22], *i.e*., overcrowding due to limited hospital bed capacity. Newly graduated nurses predominantly work in hospital settings [23], and are thus exposed to a busy work environment including seriously ill patients, often with multiple diseases and short lengths of stay. Also, the job turnover of nurses is high in many settings [24], creating a situation where the novice nurse all too soon can become 'the most experienced' at the workplace, adding to a demanding work context. It has been suggested that circumstances during early work life, such as work-related stress and a lack of experiential knowledge, hinder the provision of evidence-based practice [25,26]. The challenge that faces newly graduated nurses in clinical practice has been described as a 'reality shock' [27] or, more recently, as a 'transition shock' [28,29]. According to the authors advocating the existence of a transition shock, new nurses are confronted with the hierarchical hospital system, characterized by dominant normative behaviours, described as prescriptive, intellectually oppressive, and cognitively restrictive [29]. Previously adopted school values come into conflict with work life values, and skills such as the critical appraisal of current practices and openness to new knowledge may therefore be difficult to maintain.

In conclusion, newly graduated nurses are faced with a challenging work environment that may affect their ability to apply evidence-based practice. However, little is known about the trajectory of nurses' research use during the first years of clinical practice. Therefore, the aim of this study was to prospectively examine the extent of nurses' use of research during the first five years after undergraduate training and to specifically assess changes over time.

## Methods

### Design and participants

**Figure 1:**
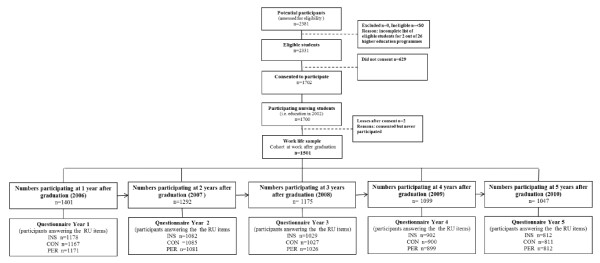
**Description of the five data collections, *i.e*., sample selection, participant recruitment, consent, timing of follow-ups and the wave response**. Work life sample (n = 1501) consisted of the group of nursing students who both entered the profession and participated in the study after education. Year 1: one year after graduation (in 2006), year 2: two years after graduation (in 2007), year 3: three years after graduation (in 2008), year 4: four years after graduation (in 2009), year 5: five years after graduation (in 2010). Abbreviations: INS = Instrumental research use, CON = Conceptual research use, PER = Persuasive research use.

Data from a prospective cohort of 1,501 Swedish newly graduated nurses within the national LANE study (Longitudinal Analyses of Nursing Education and Entry in Worklife) were used to investigate the primary outcome for this report-the extent and course of perceived use of research findings-over the first five years of practice as a nurse. Students from all of the 26 universities providing undergraduate nursing education in Sweden participated in the study, and their estimated time point for graduation was December 2004. The cohort was therefore called EX2004 (EX = examination). In total, 2,331 nursing students were invited to participate in the study while in their second semester of nursing education. 1,702 (73%) gave informed consent, and 1,501 (88%) of those subsequently also entered the profession and continued to participate in the study (Figure 1). Data were self-reported and collected through annual postal surveys. (For details of the overall LANE study, see Rudman *et al.*[30].)

The EX2004 cohort was compared with the total population of Swedish nurses who graduated in the same year to examine representativeness. Six different demographic variables from population-based national registers were tested, namely age, gender, country of birth, residency (large city), marital status, and parenthood. The only difference that was found concerned the proportion of participating females, which was 1% higher (89% versus 88%) than among all the graduating nurses in 2004 [30].

Descriptions of sample selection, participant recruitment, consent, timing of follow-ups, and wave response rates are presented in Figure 1. The 1,501 nurses who completed their undergraduate training and continued to participate in the study were assessed annually five times post-graduation-*i.e*., after one, two, three, four, and five years-and constituted the longitudinal sample to monitor change over time in the present study (in Figure 1 designated as 'the work life sample' which consisted of the group of nursing students who entered the profession as registered nurses and participated in the study after education). At data collection year one, *i.e*., after the first year post-graduation, the 1,501 nurses were on average 32.5 (SD 7.25) years old (ranging from 24 to 56 years). A majority were female (89%), of Swedish background (92%) and had previous experience in the field of healthcare (60%). The samples used for exploring extent of nurses' use of research during the first five years after undergraduate training are reported in the five boxes in the bottom of Figure 1. The discrepancy in sample size between numbers participating at one, two, three, four, and five years after graduation and the questionnaire responses year one, two, three, four, and five refers to non-responses to the specific research use items. The seemingly high discrepancies between the overall response rates and the research use response rates are related to the fact that nurses who did not work at a specific time point (for example, due to maternity or sick leave) did not fill in the work life section of the survey.

Common patterns of missing data comprised non-response in one of five data collections in work life (n = 223) and subsequent non-response after each of the first three data collections in work life (n = 239; 81 + 95 + 63 respectively). Differences between respondents with complete (across all data collections) and incomplete data were tested for age, gender, non-Swedish origin, social class, previous experience (of university studies, work in the healthcare system, or clinical training), marital status, parenthood, and self-rated health. The magnitude of these associations (*i.e*., the effect sizes) is given as estimated by tetrachoric or polyserial correlations. The associations showed that incomplete data were more frequent among younger participants (r = 0.12; *p *< 0.001), male participants (r = 0.11; *p *< 0.001), participants with non-Swedish origin (r = 0.25; *p *< 0.001), participants not raised in a working class family (r = 0.10; *p *< 0.001), and participants not entering parenthood during the first five years post-graduation (r = 0.22; *p *< 0.001). More information on the handling of missing data is presented under 'Auxiliary Variables' below.

### Instrument for measuring research use

The LANE questionnaire included three single items assessing instrumental, conceptual, and persuasive research use, originally developed by Estabrooks [16], and recently labelled as measures of 'kinds of research use' [7,17]. A Canadian version of the items published in 2004 [31] constituted the foundation for the Swedish translation and adaptation performed by our research group. Each item was structured with a definition of the concept (instrumental, conceptual, or persuasive), followed by three examples of research use exemplifying the current concept. The instrumental use item was phrased as follows:

"Instrumental research use means that you use research findings (nursing or other kinds of research) in a ***concrete ***way in providing patient care. Instrumental RU can be based on scientific articles or recommendations in systematic literature reviews, clinical guidelines, protocols or other documents based on research findings. For example:

- Assess the risk of pressure ulcers by using the modified Norton Scale.

- Use of physiological saline instead of heparin to keep a peripheral vein catheter open.

- Use of compression treatment in the treatment of venous leg ulcers."

Respondents were then asked to estimate their extent of research use according to each concept during the previous four working weeks. The response alternatives were 1 = 'never,' 2 = 'on some shifts,' 3 = 'on about half of the working shifts,' 4 = 'on more than half of the working shifts,' 5 = 'on almost every shift,' and 6 = 'don't know.' To evaluate the feasibility and face validity of the Swedish version of the items, a group of clinical nurses reviewed each item. The items were also reviewed by the staff of the technical and language laboratory at Statistics Sweden (SCB). These operations resulted in minor revisions.

The 'kinds of research use' items have been used in multiple studies with consistent findings across studies, which speak for the credibility of the measurement approach. Content validity was assessed in the index study by Estabrooks [16]. This approach to measure research use was included in a recently published systematic review on psychometric properties of research utilization instruments [17]. The report covered the following issues. Response processes (clarity and understanding of items and functionality of response scales) have been assessed in four studies and reported as valid. Significant relationships between the research use variables and other variables that theoretically or empirically have been shown to link to research use have been identified in seven studies. Further, in comparison with most other instruments assessing research use, the 'kinds of research use' include clear definitions of the constructs of interest, *i.e*., instrumental, conceptual, and persuasive use. This measure is also clear that the focus is on research use itself, not on factors related to research use, which can be confusing component of some other instruments [17].

### Analysis of longitudinal data

Descriptive cross-sectional analyses were conducted on the prevalence of research use at all five data collection waves during work life using SPSS statistics 17.0.

The longitudinal analysis applied a multilevel model (also called the linear mixed model) for change [32] implemented as latent growth curve modelling in the structural equation modelling framework [33]. The unconditional growth curve model was used to estimate a linear trajectory for the entire sample (*i.e*., estimating an intercept and a slope), at the same time estimating the amount of individual variability in baseline levels (*i.e*., variance of individual intercepts) and individual variability in the rate of change (*i.e*., variance of individual slopes). In addition, the association (*i.e*., the covariance) between baseline levels and rates of change was estimated. Moreover, the significance of an additional non-linear effect was tested, adding a quadratic effect to this unconditional growth curve model.

The latent growth curve model was estimated using the Mplus 6.0 software program [34]. In line with the current recommendations on the statistical treatment of longitudinal data [35], all available research use ratings from the 1,501 respondents were included and full information maximum likelihood estimation was used to estimate parameters in the model in the presence of missing data. In order to evaluate the stability of the estimated effects, models were re-estimated on a sample with complete research use data from all five measurement waves. Models were also estimated using the robust standard error option to correct for non-normality (*i.e*., Robust Maximum Likelihood estimator) and the categorical option to correct for the ordinal nature of the rating data (*i.e*., Robust Weighted Least Squares estimator). Before estimation, an evaluation was made of whether the clustered nature of the data needed to be taken into account (when data collection was initiated the eligible students were nested within 26 different educational institutions). The possible impact of this nesting on future research use was estimated using intraclass correlations (ICC). The correlations were generally around 0 (ICC less than 0.010 in magnitude) and the highest ICC was 0.017 (for conceptual research use one year post-graduation). Thus, these near zero effects of nesting data indicated that no further control for the impact of educational institutions was needed when estimating the effects and sources of individual differences in longitudinal growth [36].

Model fit was evaluated using multiple fit indices. These indices and proposed cut-off points were chosen on the basis of their performance in Monte Carlo simulations and recommendations based on these simulations [37,38]. Specifically, good model fit was indicated by a standardized root mean square residual (SRMR) below 0.08, a root mean square error of approximation (RMSEA) of around 0.05, a non-significant close fit test (Cfit), and a comparative fit index (CFI) of around 0.95. Misfit due to possible deviation from the linear model was explored by applying an unspecified trajectories growth model [38]. In such a model, the deviation from the imposed linear trajectory can be evaluated separately for each individual time point.

### Auxiliary variables

In order to improve the accuracy and power of the analysis, the methodological literature currently recommends the inclusion of external variables in the estimation process [35]. Such variables (called auxiliary variables) should be chosen to reflect possible differences among respondents with complete data and incomplete data (*i.e*., reflecting the assumption that data are missing at random). At the same time, these variables should not be related to levels of research use (as this would reflect an indication of data not missing at random).

As described, the comparisons between respondents with complete data across all five years versus those with incomplete data showed that non-response was more frequent among younger participants, male subjects, participants of non-Swedish origin, participants not raised in a working class family, and participants not entering parenthood during the first five years post-graduation. These variables were therefore chosen to be used as auxiliary variables. Furthermore, this set of variables was scrutinized with the aim of finding indications that data were not missing at random, *i.e*., that these variables were also related to levels of research use. Associations are given as estimated by polychoric correlations. Among these variables, only gender was associated with future research use (males using research to a lesser extent, correlation about 0.11 across measures and measurement occasions). Thus, this could introduce a bias because male subjects were also more frequently found to be among the non-responders. The methodological literature currently recommends also including such a variable, as its inclusion as an auxiliary variable will reduce-but not completely eliminate-bias in the estimation [35]. The procedure of including these external (auxiliary) variables was fully automated in the Mplus software program using Graham's saturated correlates approach [34], which has been shown to improve accuracy without altering the substantive interpretation of the parameters of the latent growth curve model [35].

### Ethical considerations

The Research Ethics Committee at Karolinska Institutet, Sweden, approved the study (KI01-045, 2001-05-14 and 2003-12-29). Initially, informed consent was provided from all respondents. They received information about the study, guaranteeing confidentiality and indicating that participation was voluntary and could be terminated at any time.

## Results

**Figure 2:**
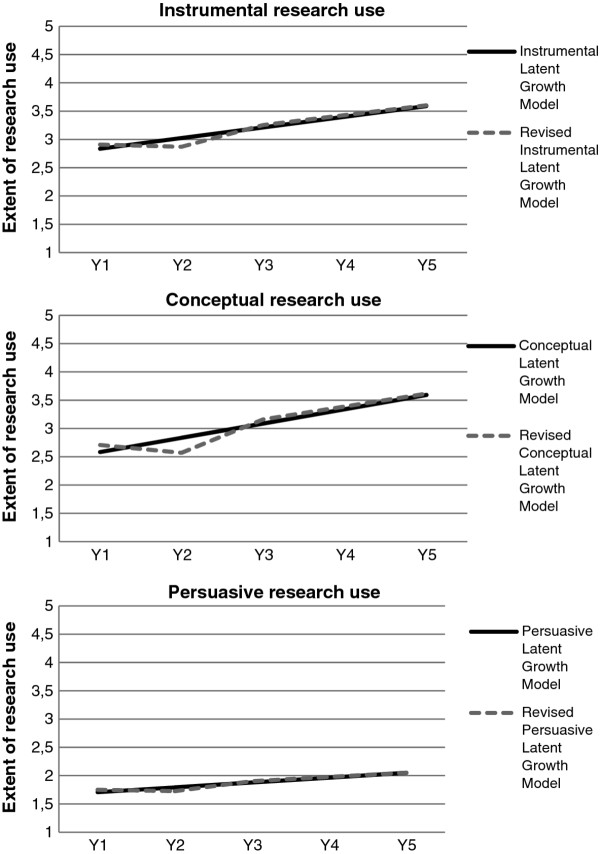
**Development of instrumental, conceptual and persuasive research use across the first five years of practice**. Estimates from a Latent Growth Model and a revised model (freeing the second time point from being included in the linear model). Extent of research use during the previous four working weeks were assessed on a response scale from 1 to 5 (the y-axis: 1 = 'never,' 2 = 'on some shifts,' 3 = 'on about half of the working shifts,' 4 = 'on more than half of the working shifts,' 5 = 'on almost every shift'). High values indicate high extent of research use and low values indicate low extent.

The findings on the nurses' ratings of the extent to which they used research in clinical practice during their first five years as nurses are first presented descriptively, focusing on the longitudinal trend of the cross-sectional material. Additionally, we make full use of prospective longitudinal data, and present results from testing linear increase over time using latent growth curve modelling. Note that the results relating to the prevalence of research use refer to the five response categories (Table 1), but in the longitudinal modelling we refer to the categories as a scale because data in that analysis is treated as continuous (Figure 2).

**Table 1:** Prevalence of research use at yearly assessments, *i.e*., one, two, three, four and five years after graduation

Instrumental		Year 1	Year 2	Year 3	Year 4	Year 5
		**n (%)**	**n (%)**	**n (%)**	**n (%)**	**n (%)**

	Never	202 (17.1)	219 (20.2)	133 (12.9)	96 (10.6)	64 (7.9)

	Some shifts	321 (27.2)	303 (28.0)	225 (21.9)	227 (25.2)	172 (21.2)

	50% of shifts	128 (10.9)	106 (9.8)	82 (8.0)	93 (10.3)	71 (8.7)

	> 50% of my shifts	136 (11.5)	104 (9.6)	106 (10.3)	104 (11.5)	83 (10.2)

	Almost every shift	264 (22.4)	263 (24.3)	376 (36.5)	316 (35.0)	353 (43.5)

	*Don't know *	*127 (10.8)*	*87 (8.0)*	*107 (10.4)*	*66 (7.3)*	*69 (8.5)*

	Total	1,178	1,082	1,029	902	812

**Conceptual**						

	Never	168 (14.4)	198 (18.2)	93 (9.1)	72 (8.0)	37 (4.6)

	Some shifts	433 (37.1)	389 (35.9)	272 (26.5)	240 (26.7)	202 (24.9)

	50% of shifts	146 (12.5)	131 (12.1)	130 (12.7)	92 (10.2)	99 (12.2)

	> 50% of shifts	110 (9.4)	102 (9.4)	121 (11.8)	112 (12.4)	109 (13.4)

	Almost every shift	160 (13.7)	136 (12.5)	327 (31.8)	306 (34.0)	281 (34.6)

	*Don't know *	*150 (12.9)*	*129 (11.9)*	*84 (8.2)*	*78 (8.7)*	*83 (10.2)*

	Total	1,167	1,085	1,027	900	811

**Persuasive**						

	Never	471 (40.2)	441 (40.8)	365 (35.6)	288 (32.0)	219 (27.0)

	Some shifts	425 (36.3)	423 (39.1)	367 (35.8)	370 (41.2)	327 (40.3)

	50% of my shifts	49 (4.2)	54 (5.0)	50 (4.9)	52 (5.8)	64 (7.9)

	> 50% of my shifts	31 (2.6)	29 (2.7)	56 (5.5)	43 (4.8)	42 (5.2)

	Almost every shift	38 (3.2)	28 (2.6)	61 (5.9)	38 (4.2)	39 (4.8)

	*Don't know *	*157 (13.4)*	*106 (9.8)*	*127 (12.4)*	*108 (12.0)*	*121 (14.9)*

	Total	1,171	1,081	1,026	899	812

### Five years' longitudinal data on nurses' extent of research use

There was variation in the extent of the three 'kinds of research use' (Table 1). Overall, instrumental use of research was the most frequently reported, but there was considerably less difference between instrumental and conceptual use than between instrumental/conceptual and persuasive use of research. Taking findings from the data collection wave five years after graduation as an example, 44% of the nurses rated their instrumental use of research as occurring 'on almost all shifts,' 35% reported conceptual use, and 5% of the respondents reported persuasive use of research 'on almost all shifts.' On the other hand, also using year five data, 67% of the respondents reported infrequent use of persuasive research (*i.e*., 'not at all' and 'on some shifts'), compared to 29% for both instrumental and conceptual use of research.

### Instrumental research use

Focusing on instrumental use, the cross-sectional findings over the five years showed that the nurses' use of research initially looked stable and, if anything, lessened as indicated by a small dip (Table 1). In the first and second years, a similar proportion of the nurses (34%) reported that they used research instrumentally on 50% or more of their shifts, while 44% of them rated 'never' and 'on some shifts' in year one and 48% in year two. However, in year three there was a rise in frequent users ('50% and above') to 47% of the nurses. This upward trend continued through year five, when 54% of the nurses reported their instrumental use to be this frequent. The infrequent research users ('never' and 'on some shifts') changed from representing 44% of the sample in year two to 29% in year five.

Treating respondents' ratings as continuous data and imposing a linear growth model across the five years of data resulted in a baseline (year one) mean of 2.8 and an unstandardized linear slope of +0.19 (Figure 2a and Table 2). The model showed good fit (Table 2) and illustrates the finding that nurses went from a moderate level of instrumental research use (a mean of 2.8) to increase their research use (mean of about 3.5) at year five (see Stability of results below for more information on model fit). The model also shows that there was a substantial variability in research use at year one and modest (but still significant) variability in individuals' increase over time. This means that the variability was initially large relative to the variability in change. In addition, there was a correlation between initial levels and change over time, reflecting that the rate of increase was smaller for those with an initially high level of research use. Furthermore, the possible presence of a non-linear effect was tested by adding a quadratic main effect to the growth model. The *χ*^2 ^did not decrease significantly and the new added quadratic effect was not statistically significant. Because this new parameter did not significantly improve model fit, no further parameters were added to the model.

**Table 2:** Estimates and model fit from the latent growth curve model

		Evaluation of model fit				Longitudinal main effects			Variability	
**Estimator**		***χ*^2§^**	**CFI**	**RMSEA (C)**	**SRMR**	**Intercept**	**Slope**	**Cov (I, S)**	**Var (I)**	**Var (S)**

***Instrumental research use ***	**ML^a ^**	47.3***	0.95	0.05 (.48)	0.06	2.84***	0.19***	-0.09**	0.99***	0.06***

	**ML^a, b ^**	46.2***	0.95	0.05 (.37)	0.06	2.86***	0.14**	-0.09**	1.00***	0.06***

	**MLR^a ^**	44.7***	0.94	0.05 (.56)	0.06	2.84***	0.19***	-0.09**	0.99***	0.06***

	**WLS^a ^**	97.2***	0.95	0.06 (.16)	-	0	0.14**	-0.04**	0.52***	0.03***

	**ML^c ^**	43.4***	0.92	0.08 (.02)	0.08	2.92***	0.18***	-0.07 ns	0.95***	0.05**

	**ML^a, d ^**	24.4**	0.98	0.03 (.95)	0.05	2.91***	0.17***	-0.05**	0.95***	0.04***

***Conceptual research use ***	**ML^a ^**	110.1***	0.72	0.08 (.01)	0.06	2.58***	0.25***	-0.07**	0.61***	0.05***

	**ML^a, b ^**	109.2***	0.72	0.09 (.01)	0.06	2.57***	0.29***	-0.07**	0.60***	0.05***

	**MLR^a ^**	105.4***	0.71	0.08 (.01)	0.06	2.58***	0.25***	-0.07**	0.61***	0.05***

	**WLS^a ^**	209.0***	0.67	0.09 (.01)	-	0	0.21***	-0.05**	0.41***	0.04***

	**ML^c ^**	70.7***	0.76	0.11 (.01)	0.08	2.58***	0.26***	-0.09*	0.69***	0.06***

	**ML^a, d ^**	41.1***	0.91	0.05 (.52)	0.04	2.71***	0.23***	-0.03 ns	0.52***	0.03***

***Persuasive research use ***	**ML^a ^**	31.2***	0.96	0.04 (.96)	0.04	1.71***	0.09***	0.01 ns	0.29***	0.02**

	**ML^a, b ^**	31.0***	0.96	0.04 (.83)	0.04	1.71***	0.07*	0.01 ns	0.29***	0.02**

	**MLR^a ^**	24.2**	0.96	0.03 (.98)	0.04	1.71***	0.09***	0.01 ns	0.29***	0.02**

	**WLS^a ^**	39.2**	0.99	0.03 (.99)	-	0	0.10***	-0.02 ns	0.48***	0.02**

	**ML^c ^**	14.6 ns	0.99	0.02 (.99)	0.04	1.76***	0.10***	0.01 ns	0.32***	0.02**

	**ML^a, d ^**	15.3 ns	0.99	0.02 (.99)	0.03	1.75***	0.08***	0.01 ns	0.29***	0.02**

^a ^Estimation includes all available data from any of the five data collections.

^b ^Model includes an additional quadratic main effect.

^c ^Estimation includes only those with complete RU data from all five data collections.

^d ^Revised model, freeing the second time point from being included in the linear model.

^§ ^All models have 10° of freedom except the model with a quadratic effect (df = 9), the WLS estimated model (df = 18), and the revised model (df = 9).

Abbreviations: *ML *= Maximum Likelihood; *MLR *= Robust Maximum Likelihood; *WLS *= Weighted Least Square (note that SRMR are not computed with this estimator and that estimated effects are on another scale); *CFI *= Comparative Fit Index; *RMSEA *= Root Mean Square Error of Approximation; (C) = *p*-value for RMSEA deviating from 0.05; *Cov (I, S) *= covariance between variability in intercept and slope; *Var (I) *= intercept variance; *Var (S) *= slope variance; ns = non significant; *p < .05; **p < .01; ***p < .001.

### Conceptual research use

Conceptual research use exhibited a pattern similar to instrumental use, but at a somewhat lower initial level (Table 1). There was also a slight decrease in research use between years one and two. One year after graduation, 23% of the nurses reported conceptual use of research on '50% and above' of their shifts, compared to 21% after two years. Analogous to instrumental use, an increase in conceptual use was reported at year three. Three years after graduation, 40% of the nurses rated their conceptual use of research as occurring on '50% and above' of their work shifts. At year five, this higher level of conceptual research use was reported by 45% of the nurses. Parallel to this, the low users' ratings changed from representing 54% of the sample in year two to 29% of the sample in year five.

Applying the linear growth model to nurses' ratings of conceptual research use generated a mean of 2.6 at year one and an unstandardized linear slope of +0.25 (Figure 2b and Table 2). From a moderate level (a mean of 2.6), the conceptual use of research increased to a mean of 3.6 at year five (see Stability of Results below for more information on model fit). Similar to the model of instrumental research use, this model showed a substantial variability in conceptual research use at year one, but only modest variability of individuals' increase of ratings over time. Also similar to the instrumental model, the rate of increase was smaller for those with an initially high level of research use. However, these data must be interpreted with caution because two indices of model fit indicated poor model fit (see below for an exploration of causes of misfit). Finally, the inclusion of a quadratic effect did not improve model fit, and no further parameters were added to the model.

### Persuasive research use

It was also possible to identify a pattern of a small dip in persuasive research use from year one to year two, and an incremental increase thereafter, albeit at a substantially lower level compared with instrumental and conceptual use (Table 1). Persuasive research use on '50% and above' of the work shifts was stable between years one and two, comprising about 5% of the nurses. At year three, persuasive research use had changed to comprise 11% of the nurses, and this figure remained at a similar level (10%) at year five. The proportion of nurses rating their persuasive use of research at the lower end of the response scale ('never' and 'on some shifts') started with 77% of the nurses at year one and ended at 67% in year five.

Applying the linear growth model to the data on persuasive research use resulted in a year one mean of 1.7 and an unstandardized linear slope of +0.09 (Figure 2c and Table 2). Similar to the instrumental model, this model showed good model fit (Table 2). From a modest level of using research persuasively (a mean of 1.7 in year one), the nurses reported a modest increase in their persuasive use ending at a mean of about 2.0 at year five (see Stability of Results below for more information on model fit). Similar to the other two models, this model indicated a large variability at year one but a smaller variability in the increase of research use in individuals over time. Again, an inclusion of a quadratic effect did not improve model fit, and no further parameters were added to the model.

### Stability of results

In order to evaluate the longitudinal results, the linear growth model was also estimated with other estimators (MLR and WLS) and applied to a smaller sub-sample comprising only those individuals with complete ratings of research use from all data collection waves. As can be seen in Table 2, model fit and estimated parameters were almost identical regardless of estimator used. In addition, the longitudinal effects estimated for the smaller sample are close, in fact almost identical, to estimates from the larger sample. When using this smaller sub-sample, the only differences that can be observed concern significance levels due to the loss of power.

An exploration of causes of misfit in the longitudinal model for the conceptual research use data was undertaken applying an unspecified trajectories growth model and revealed that ratings from the second year deviated from the linear trend. Not imposing a linear trend between the two first years resulted in a large improvement in model fit (Table 2 and Figure 2) The estimated linear trend (when freeing the second time point from being included in the linear model) generated a mean of 2.71 at year one and a linear slope of +0.23. Similar to the original model, this model indicated a substantial variability in conceptual research use at year one but a modest variability of individuals' increase of ratings over time. The same revised model was also applied to the longitudinal data for Instrumental research use and Persuasive research use (Table 2 and Figure 2). In similar, there was a 50% reduction in *χ*^2 ^and an improvement in model fit.

## Discussion

In this five-year longitudinal study, we found instrumental use of research to be most frequently reported, closely followed by conceptual use. The development over time showed a consistent pattern of upward trend for all three 'kinds of research use,' but with the descriptive findings indicating that the increase in using research only started after the second year. Using the same metric approach as Squires and co-workers [7], *i.e*., dividing the possible range of scores into quartiles: low (1.0 to 1.99), moderate-low (2.0 to 2.99), moderate-high (3.0 to 3.99) and high (4.0 to 5.0), we found that instrumental and conceptual use went from moderate-low in year one to moderate-high in year five, and persuasive use from low in year one to moderate-low in year five. The extent of research use at year five is comparable to what has been reported in other studies using the same measures [7]. One issue that may be of more interest is whether these levels of research use are 'acceptable.' One could expect that nurses should use research findings on all working shifts (at least instrumentally). For example, presumably nurses should wash their hands between patients on all shifts. However, it must be noted that the items measure the perceived extent of research use, making the scoring dependent on the respondents' awareness of the knowledge base of their clinical practice. This makes it hard to obtain a firm estimation of what levels of research use could optimally be expected.

Several authors have emphasized the importance of successful adjustment to work life for nurses' professional development as well as for quality of patient care [39,40]. Our findings indicate that there was a 'delay,' extending over two years, in the development of professional practice in terms of research use, which might be a manifestation of a 'transition shock.' Using patterns across all three types of research use to address extent of research use, our group previously identified an increase in the number of individuals characterized by a profile of low use of research from year one to year two after graduation [6]. Consequences of a stressful entry into work life, such as burnout, have frequently been described in the literature [5,29,30,41,42]. The underlying causes may be that the new nurses invest a great deal of time and energy in their professional role in order to manage the situation and adjust for their inexperience. The burden of work, in combination with novice nurses' limited skills, may drain their energy further and result in a downward prioritization of certain work tasks or even lowered ambitions. A recent study on the development of burnout among nurses during the first three years post-graduation showed that the most typical change trend was substantial increases in burnout levels between the first and second years of practice [43]. As symptoms of stress, like job burnout, have been previously shown to be related to lower research use [44,45], the initial stable low level of research use found in the present study may be a consequence of the newcomers' struggle to handle their inexperience in the intensive and challenging reality of work life.

There was a significant linear increase in all three kinds of research use. This was most prominent for conceptual use, which went from a mean of 2.6 in year one to 3.6 in year five. The reason for this change cannot be fully understood from our data. It could be interpreted in at least two ways. There appears to be a beneficial adaptation to contextual conditions that starts to have an effect after two years, and there are factors in the work context that support this development. The work context comprises plenty of factors that may support or hinder the use of research-based knowledge, such as the behaviour of leaders, evaluation and feedback mechanisms, professional interaction, and the availability of information sources such as research databases and practice guidelines [46,47]. Such components of context may of course facilitate positive development. This would also imply that nursing education does not prepare all nurses sufficiently to use research in a deliberate way; this skill appears to be acquired gradually during professional life. Our findings might also be interpreted in the light of Benner's application of the Dreyfus model of skill acquisition to nursing [48]. In this model, nurses develop from being novices and advanced beginners to become competent clinicians after two to three years of clinical experience. At this more competent stage of practice, nurses have developed from rule-governed decision-making to the conscious, abstract, analytical contemplation of clinical problems, which could include increased awareness and use of research to guide practice.

Additionally, these findings provide some food for thought on the issue of individual characteristics versus contextual factors related to evidence-based practice. Rycroft-Malone argues for not viewing evidence use as an individual activity; instead, it would be more helpful to see evidence-based practice as a system property acknowledging the importance of context [49]. We also believe that such a perspective is beneficial for obtaining a better understanding of how research is used in practice. However, our findings indicate that individual characteristics are important ingredients in establishing evidence-based practice. There was a substantial degree of individual variability in research use levels at year one (which was most prominent for instrumental use), but a more modest variability among the respondents in the amount of increase over time. This means that the levels of research use at year one-proximal to undergraduate training-is an important indicator for the level of research use over the first five years. It underlines that individual characteristics, such as attitudes, intentions, skills, and knowledge, which students bring from undergraduate training into work life, play a substantial role in terms of their use of research in their early professional careers. This is in line with the findings of Squires *et al.*[7]; attitudes towards research were a determinant of instrumental research use found in four studies using the 'kinds of research use' measure, and additionally found as a determinant in eight studies examining research use in general. It also implies that undergraduate education, at least to some extent, did prepare the nurses to be eventual users of research in their practice.

### Methodological considerations

The current study has some obvious strengths. The data cover five consecutive years early on in the subjects' professional careers and constitutes, to the best of our knowledge, the first prospective longitudinal study on nurses' use of research. We have a national sample with good response rates throughout the five data collection waves. The outcome measure-'kinds of research use'-has been the subject of psychometric evaluation and judged to have a number of assets concerning validity [17]. However, there are issues with measuring research use through self-report. The main problem is that a respondent will base the ratings of extent of research use on what he/she is aware of being research-based, rather than actual use of research. Another limitation is the categorical response scale, causing a lack of precision in the measurement.

In the present study, latent growth curve models were utilized, but analyses of possible causes of inadequate fit indicated that levels of conceptual research use did not show a linear increase from baseline to the second year. A lack of increase, or even a decrease, of conceptual research use appeared when scrutinizing the extent of high and low users in the cross-sectional data. Because this trend was also apparent for the other two dimensions of research use, the same exploratory model was applied as for the conceptual research use data. Again, model fit increased (from good to excellent) when allowing data from the second year to deviate from the linear increasing trend. This non-linear trend could of course reflect idiosyncratic characteristics of our sample, and replications are needed before drawing firm conclusions based on this trend. However, as previously discussed, the first two to three years post-graduation can be very demanding for newly graduated nurses.

There was, as expected, a successive loss of respondents over the five years, but our analyses do not indicate that this would have threatened the internal validity of the study. However, males were more often found among non-respondents and at the same time were also found to report low research use more often. This raises an issue: can the identified trend of increasing research use over the first years of professional life be an indication of a selection bias reflecting that low users leave the cohort? First, the subsample of male subjects is small (less than 10%) and the correlation between gender and response across time is very low (r = 0.11), so this interaction would not produce the longitudinal trends found for research use. Moreover, when all longitudinal data analyses were estimated on both a sample using all available data and on a (possible selective) sample only including respondents with complete data from all data collections, estimated parameters were found to be almost identical in the two samples. Taken together, the stable results, with replication in the two samples, suggest that the longitudinal effects observed in this study are valid.

## Conclusion

There was a clear trend of increasing research use by nurses during their first five years of practice. This trend of increase, encompassing all three kinds of use, and a relatively small amount of individual variability in the increase, indicates that the initial level of research use is an important indicator of the level of research use over the first five years. What the students bring from undergraduate training into work life appears to play a substantial role in terms of their use of research in their early professional careers. However, it must be noted that it took more than two years of professional development before this increase 'kicked in.' This 'delay' supports previous research claiming that newly graduated nurses go through a 'transition shock' that, at least initially, reduces their ability to use research findings in clinical work. Our findings emphasize the need to consider both individual characteristics and contextual factors in the promotion of evidence-based practice.

## Competing interests

The authors declare that they have no competing interests.

## Authors' contributions

LW contributed to the design of the study and drafted the manuscript. PG contributed to the design of the study, performed the statistical analysis, and helped to draft the manuscript. AE contributed to the design of the study and helped to draft the manuscript. AR contributed to the design of the study, was responsible for acquisition of data, performed the statistical analysis, and helped to draft the manuscript. All authors read and approved the final manuscript.
